# The Role of CD4 T Cell Memory in Generating Protective Immunity to Novel and Potentially Pandemic Strains of Influenza

**DOI:** 10.3389/fimmu.2016.00010

**Published:** 2016-01-25

**Authors:** Anthony DiPiazza, Katherine A. Richards, Zackery A. G. Knowlden, Jennifer L. Nayak, Andrea J. Sant

**Affiliations:** ^1^Department of Microbiology and Immunology, David H. Smith Center for Vaccine Biology and Immunology, University of Rochester Medical Center, Rochester, NY, USA; ^2^Department of Pediatrics, David H. Smith Center for Vaccine Biology and Immunology, University of Rochester Medical Center, Rochester, NY, USA

**Keywords:** CD4 T cells, influenza, human, vaccines, antibodies, memory

## Abstract

Recent events have made it clear that potentially pandemic strains of influenza regularly pose a threat to human populations. Therefore, it is essential that we develop better strategies to enhance vaccine design and evaluation to predict those that will be poor responders to vaccination and to identify those that are at particular risk of disease-associated complications following infection. Animal models have revealed the discrete functions that CD4 T cells play in developing immune response and to influenza immunity. However, humans have a complex immunological history with influenza through periodic infection and vaccination with seasonal variants, leading to the establishment of heterogeneous memory populations of CD4 T cells that participate in subsequent responses. The continual evolution of the influenza-specific CD4 T cell repertoire involves both specificity and function and overlays other restrictions on CD4 T cell activity derived from viral antigen handling and MHC class II:peptide epitope display. Together, these complexities in the influenza-specific CD4 T cell repertoire constitute a formidable obstacle to predicting protective immune response to potentially pandemic strains of influenza and in devising optimal vaccine strategies to potentiate these responses. We suggest that more precise efforts to identify and enumerate both the positive and negative contributors within the CD4 T cell compartment will aid significantly in the achievement of these goals.

## The Specificity of Human CD4 T Cells to Influenza Virus

**Figure 1:**
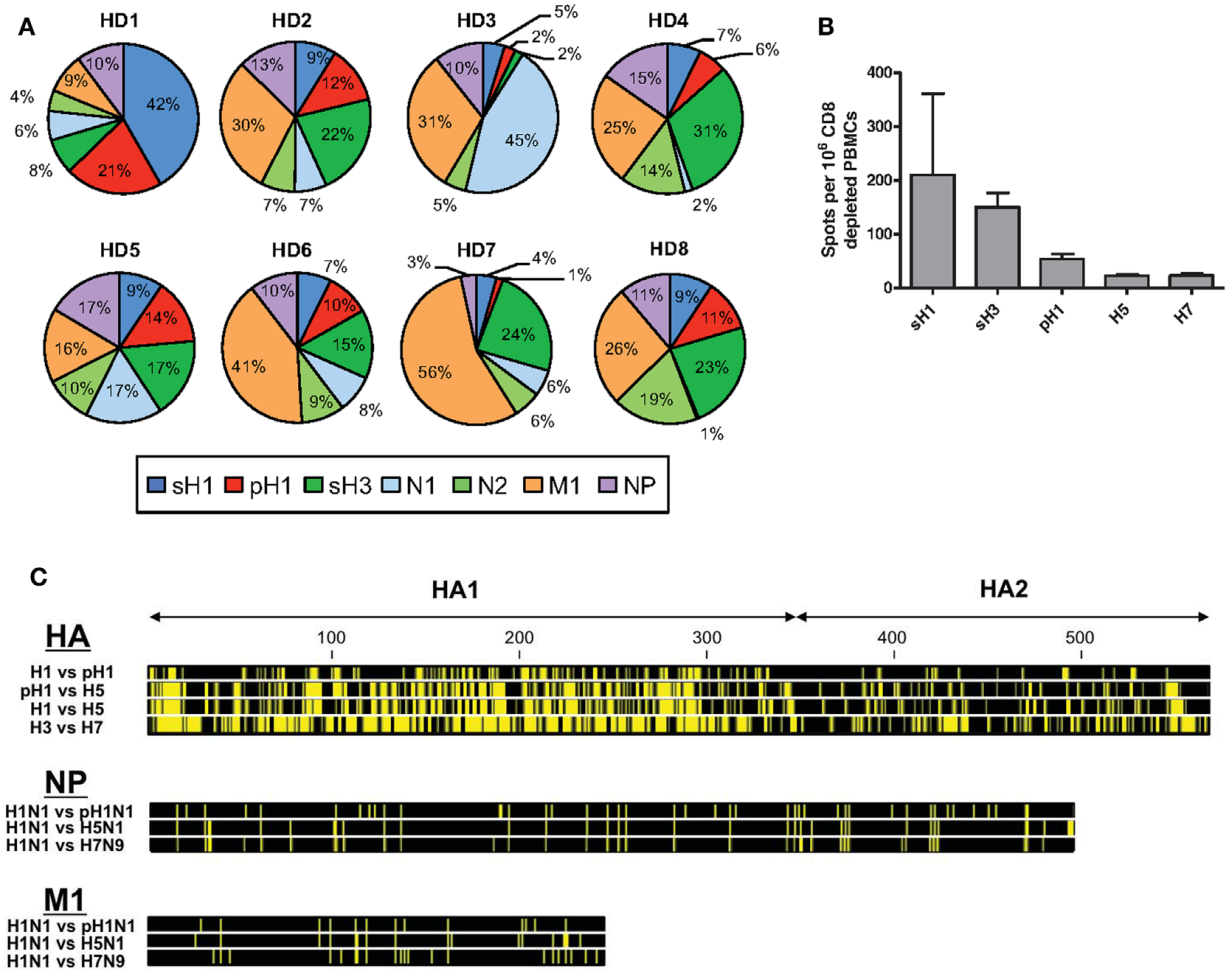
**Patterns of human CD4 T cell reactivity to influenza viral proteins in healthy subjects**. In **(A)**, IFN-γ Elispots were used to enumerate antigen-reactive CD4 T cells. CD4 T cells isolated from eight healthy adults were tested for reactivity to individual proteins using overlapping peptide pools representing the entire translated sequence of the indicated influenza protein. The relative reactivity to each protein is represented in a pie diagram, with each viral protein indicated by a different color and the percent of the total influenza response in the different subjects indicated by the size of the slice. In **(B)**, CD4 T cell reactivity to different seasonal and pandemic HA peptide pools were compared. Reactivity to pH1N1 HA protein was determined using the pre-vaccination reactivity of 49 healthy adult donors (9, 13). Reactivity to avian proteins was determined using baseline reactivity of a panel of 30 (H5) and 23 (H7) seronegative donors, enrolled as part of pandemic vaccine studies (12). **(C)** shows the amino acid sequence conservation for viral HA, NP, and M1 proteins. Yellow bars represent segments of sequence variation and black segments denote stretches of sequence identity at each position. Viral isolates for each HA subtype are as follows: H1 – A/Brisbane/59/2007 (H1N1), pH1 – *A/California/07/2009 (H1N1)*, H5 – *A/Vietnam/1203/2004* (H5N1), H3 – A/Texas/50/2012 (H3N2), and H7 – *A/Anhui/1/2013 (H7N9)*, where italics indicate divergent viruses with pandemic potential to humans. Viral isolates for NP and M1 are as follows: H1N1 – A/Puerto Rico/8/1934, pH1N1 – *A/California/07/2009*, H5N1 – *A/Vietnam/PEV16T/2005*, and H7N9 – *A/Anhui/1/2013*. Sequence files were downloaded from PubMed and conservation profiles were constructed using CLC Sequence Viewer 7 software.

It has become increasingly clear that CD4 T cell immunity to influenza has broad specificity (1–4). CD4 T cells specific for influenza viral proteins can be readily detected in the circulation of most human subjects, when assayed through approaches, such as HLA-class II tetramer staining (5), intracellular cytokine staining (6), and cytokine Elispots (7). Our laboratory has used cytokine Elispots and large peptide libraries to assess the viral protein specificity of influenza-specific CD4 T cells in an unbiased approach (8, 9). Figure 1A shows the results from the sampling of CD4 T cells isolated from PBMC of healthy adults for reactivity to influenza viral proteins, including hemagglutinin (H1 and H3), neuraminidase (N1 and N2), nucleoprotein (NP), and matrix 1 (M1) from seasonal isolates (8). These studies have revealed abundant CD4 T cell reactivity to most influenza viral proteins, with variable frequency, typically in the range of 0.03–0.4% of the total circulating pool when all specificities are summed. This finding of a broad CD4 T cell repertoire to influenza is in agreement with our studies in animal models that show that CD4 T cells elicited in response to infection (4, 8, 10) and vaccination (11, 12) include specificity for almost all viral proteins, depending on the host’s MHC class II proteins. Humans have many options for HLA class II molecules to present peptides to CD4 T cells due to multiple isotypes (HLA-DP, HLA-DQ, and HLA-DR), their codominant expression and heterozygosity. Therefore, their CD4 T cell repertoire is likely broader than inbred mice. This diversity of influenza-specific CD4 T cells in humans has raised intriguing issues and challenges relevant for predicting vaccine responses and protection from influenza infection.

## The Multiplicity of Functions of Influenza-Specific CD4 T Cells

Many functions of influenza-specific CD4 T cells have been described (14–17). Collectively, these studies have revealed that CD4 T cells contribute in diverse ways and at different sites *in vivo* to protective immunity to influenza. CD4 T cells provide essential help for high-affinity, neutralizing antibody responses, an activity conveyed by CD4 T follicular helper cells (Tfh) within the germinal centers of secondary lymphoid organs (18–22). Within the draining lymph node, CD4 T cells can also enhance the recruitment of other effector cells, facilitate engagement of CD8 T cells with dendritic cells, and promote CD8 T cell priming and memory. Moreover, CD4 T cells can engage in direct cytotoxicity of antigen bearing cells, a function suggested to be the primary correlate of protection from infection in humans (23). Finally, within the lung, memory CD4 T cells provide diverse functions including production of antiviral cytokines, such as IFN-γ, promotion of early recruitment of innate effectors, and potentiation of CD8 T cell recruitment, localization, and persistence (24–26).

This multiplicity of potential functions contributed by memory CD4 T cells, each conferred by distinct arrays of soluble mediators and cell surface proteins, presents a significant challenge for predicting and enhancing protective immunity to potentially pandemic strains of avian influenza*. In our view, several key questions need to be resolved in order to better understand the impact of CD4 T cells on human immunity to influenza infection*. The first is whether different specificities of influenza-reactive CD4 T cells convey different functions. We hypothesize and have supporting evidence that the functional potential of CD4 T cells may be linked to both the fine specificity and the viral protein of origin. Second, under what circumstances are particular effector functions of CD4 T cells the *limiting factor* in the protective response? These limiting functions would be those that need to be monitored in susceptible hosts pre- and postinfection and enhanced by vaccination. Finally, to what degree do the different CD4 T cell subsets and their potentially unique specificities regulate each other’s function and how much do these interactions confound efforts to quantify the contribution of CD4 T cells to influenza immunity? We will discuss these issues and our own work that sheds light on them below.

## Links Between Specificity and Function of CD4 T Cells in Influenza

Because of the importance of neutralizing antibodies in protection from influenza, we have explored the role of viral protein specificity in provision of CD4 T cell help for antibody responses to vaccines and infection. Several studies have shown that Tfh cells can be a limiting factor in the B cell response (27–29). We used a mouse model utilizing synthetic peptides (previously identified to be co-immunodominant) to generate CD4 T cell memory independently of B cell activation. These studies revealed an inseparable linkage of specificity in the provision of CD4 T cell help to antigen-specific B cells (30), a result in agreement with earlier studies using vaccinia virus (31). We found that mice with CD4 memory to NP demonstrated an enhanced antibody response to NP, but not HA, while those with CD4 T cell memory to HA exhibited an accelerated antibody response to HA, a phenotype associated with lower viral titers in the lungs. We interpret this important result to mean that HA-specific memory CD4 T cells can potentiate early neutralizing antibody production that can diminish the yield of infectious virus.

Our studies of the human response to influenza vaccination agree with and extend this concept of linked specificity to vaccination. Although licensed vaccines are quantified only for HA from the manufacturers, inactivated vaccines produced in embryonated chicken eggs also contain the membrane protein NA and internal viral proteins, such as M1 and NP (32, 33). The presence of these additional viral proteins has been detected by both biochemical and functional assays. Therefore, these vaccines will recruit CD4 T cells specific for many viral proteins, some of which are novel (i.e. HA and NA) and some conserved (i.e. NP and M1). The consequences of boosted memory CD4 T cells competing with naïve CD4 T cells specific for novel epitopes within HA and NA is not known, nor do we understand if all CD4 T cells elicited by the vaccine will promote production of protective antibodies. In two separate studies, we have tracked the expansion of human CD4 T cells after vaccination, using cytokine Elispots and large peptide pools derived from discrete viral proteins that bypass the need for antigen processing. When CD4 T cell responses were tracked over time, we found that expansion of CD4 T cells specific for peptide epitopes within HA, but not NP, positively correlated with the neutralizing anti-HA antibody response (9, 12). We speculate that the form of antigen taken up by HA-specific B cells after vaccination and after infection does not include NP. According to this model, the specificity of recruited CD4 T cells to facilitate the neutralizing antibody response is dependent on the nature of the antigen internalized by HA-specific B cells.

We have also found an intriguing difference between the functional potential of HA- and NP-specific CD4 cells through the analyses of specificity in “Tfh-like” cells and non-Tfh cells in the circulation of healthy adults. Antigen-experienced CD4 T cells were separated based on expression of the prototypic marker of Tfh, CXCR5, a chemokine receptor shown to be expressed on a subset of circulating human CD4 T cells that likely represents circulating memory Tfh (34–36). We (37) and others (38) found that CXCR5^+^ CD4 T cells were selectively able to promote antibody production when cocultured with naïve B cells (36). Interestingly, when examined for influenza specificity, CXCR5^+^ Tfh-like cells were enriched for reactivity to HA, whereas CXCR5^−^ non-Tfh cells were preferentially reactive to NP. This pattern, detected by both cytokine Elispots and a cytokine-independent assay, was observed in multiple individuals, each presumably with a distinct history of infection and vaccination. Collectively, these results suggest that HA-specific CD4 T cells likely have the most potential to provide help for neutralizing antibody responses and may be the most critical to monitor pre- and post-vaccination. CD4 T cells specific for other viral proteins, such as NP, may not only fail to provide help but may also be preferentially associated with alternative effector programs, including expression of IFN-γ and CXCR3.

## The Challenge to Successful Vaccination and Protection from Novel, Pandemic Strains of Influenza

The preceding findings regarding the link between HA specificity of CD4 T cells and antibody responses are of particular importance when considering the ability of circulating CD4 memory to provide help for protection against novel and potentially pandemic strains of influenza. Shown in Figure 1C is the comparison of the extent of sequence identity between influenza proteins derived from seasonal strains and potentially pandemic strains (pH1N1, H5N1, and H7N9), where yellow represents divergence of the potentially pandemic strain from the indicated seasonal strain. It is clear from this comparison that M1 and NP offer more regions of sequence identity than does HA and thus more potential for cross-reactive CD4 T cell recognition of potential pandemic strains. Others have identified such cross-reactive CD4 T cells (39–42). However, our results, thus far, suggest that many of these CD4 T cells, specific for internal virion proteins, may not contribute positively to the antibody response following infection or vaccination.

It is our view that for provision of help for neutralizing antibody responses to pandemic influenza, HA-specific cells may be the most critical. In the host that has not previously encountered a new and potentially pandemic strain of influenza, these HA-specific CD4 T cells must be drawn from the memory pool elicited previously by seasonal strains. We have found that the relatively small pool of HA-specific memory CD4 cells that can be recruited into the response to novel strains of influenza will vary among individuals, likely reflecting their exposure history with seasonal influenza. The potential for cross reactive recognition by HA specific CD4 T cells will also depend on the relatedness between the HA proteins, clear from the comparison of Figures 1B,C. For example, the pandemic strain of influenza (“pH1N1”) that emerged in 2009 expressed a novel H1 HA protein originally encoded within a classic swine influenza strain (43–46). Because this HA was an H1 protein, there was significant (79%) sequence identity with recently circulating seasonal H1 HA proteins. In agreement with this, using a murine model, we demonstrated that infection with seasonal H1N1 established substantial numbers of memory CD4 T cells that could be mobilized and provide protection upon infection with the pH1N1 strain (47). We (9) and others (48, 49) have also shown that vaccination of humans with a monovalent pH1N1 vaccine successfully elicited neutralizing antibody responses despite very little B cell cross-reactivity, suggesting that the memory CD4 T cells were able to facilitate a primary B cell response to unique pH1 epitopes. The pH1N1 vaccine elicited a robust HA-specific CD4 T cell response primarily drawn from memory CD4 T cells specific for peptides shared between the seasonal and pandemic strain. Moreover, expansion of CD4 T cells specific for these conserved HA epitopes was the best CD4 T cell correlate of the neutralizing antibody response (9). We suspect that this greater degree of CD4 T cell cross-reactivity may be responsible for the better antibody response to pH1N1 vaccination compared to avian influenza-derived vaccines and may also have served to temper disease progression during the initial spread of this virus.

The challenge for provision of CD4 T cell help for antibody responses to novel strains of influenza is much more profound for avian strains than the pH1N1 2009 strain. Numerous reports have documented the “poor immunogenicity” of avian-derived influenza vaccines (50–52) unless administered at high doses or with adjuvants (53, 54). The reasons underlying these vaccine failures are not completely clear, but it seems likely that this is in part due to the lack of cross-reactive B and CD4 T cell memory. It has been well documented that memory T and B cells are preferentially recruited into immune responses (55–57). These advantages likely reflect greater numbers of lymphocytes, lower TcR signaling thresholds as well as less reliance on the costimulatory signals needed for initial activation of naïve lymphocytes. These factors collectively lead to their preferential recruitment into an immune response to pathogens or vaccines that simultaneously present both conserved and novel epitopes to the immune system. In fact, the presence of responses to conserved epitopes can antagonize the clonal expansion of naïve CD4 T cells specific for novel epitopes (13).

In an effort to understand the potential for seasonal influenza to establish memory CD4 T cells capable of being recruited into a subsequent response with avian H7 HA-derived CD4 T cell epitopes, we surveyed human subjects with no history of encounter with H7 viruses or vaccines. PBMC were isolated from these healthy adults, each with presumably distinct and complex immunological history with seasonal influenza. Among the 20 healthy adults analyzed, approximately 60% had readily detectable CD4 T cell reactivity to peptides derived from the H7 HA protein and the vast majority of CD4 T cell reactivity was focused on epitopes contained within the carboxy-terminal half of the H7 protein (58). In individuals possessing CD4 T cells that cross-reacted with H7, reactivity was focused on several “hot spots” of the HA2 domain that represent some, but not all, of the regions of high sequence identity between H7 and seasonal HA, in particular H3. We concluded from these studies that memory CD4 T cells elicited by seasonal influenza can be recruited by H7-derived epitopes and are primarily drawn from epitopes in the seasonal strain that is the closest phylogenetic relative.

## How Do We Construct a Tally Sheet That Will Predict the Impact of Memory CD4 T Cells to Contribute to Protective Immunity to Influenza?

Existing data suggest that routine encounter with seasonal influenza through infection or vaccination regularly boosts CD4 T cells of many specificities, establishing memory cells that can cross-reactively recognize epitopes specific from potentially pandemic strains. These circulating influenza-reactive CD4 T cells are heterogeneous with regard to expression of chemokine receptors and the gene expression profiles linked to distinct effector functions. Only a fraction of these memory CD4 T cells have the specificity and effector characteristics needed to promote neutralizing antibody production. Therefore, only a subset of these circulating CD4 T cells can facilitate neutralizing antibody responses to the pandemic strain of influenza, while others either might not be recruited to the germinal center response at all or if they are, might antagonize development of the needed effector function. Antagonism of help by memory CD4 T cells of other specificities could be due to production of soluble inhibitors, such as IL-10, TGF-β, and IFN-γ. As influenza-specific CD4 T cells often have a dominated T helper 1 phenotype, IFN-γ is a prominent effector cytokine produced in response to infection. In addition to its proinflammatory activities, IFN-γ has a well-characterized role in inducing the production of indoleamine 2,3-dioxygenase (IDO) in dendritic cells and other antigen-presenting cells. IDO in turn can promote tryptophan degradation, which can cause T cell apoptosis, diminish T cell proliferation, or through the by-products of tryptophan metabolism, can have many antagonistic effects (59–61). Although the complex regulatory pathways induced by IDO have largely been explored in the context of tumor-specific immunity and chronic infections, IFN-γ and IDO have been implicated in suppressing responses to influenza (62). Thus, cytokines produced by memory CD4 T cells have the potential to induce immunosuppressive pathways that can directly inhibit CD4 T cell expansion or act through induction of tolerogenic dendritic cells and conventional CD4 T regulatory cells. Inhibition might therefore occur at the initial CD4 T cell-priming event needed to activate Tfh cells or by active suppression of the germinal center response, through the participation of T follicular regulatory cells [reviewed in Ref. (63)].

Many questions arise from this model that seeks to quantify and predict the impact of memory CD4 on influenza immunity. If CD4 T cell help in the germinal center response is a major factor limiting the magnitude of the neutralizing antibody response, how many CD4 T cells are needed and what fraction of the circulating memory will be recruited into response to vaccination? It is clear that only a subset of circulating CD4 T cells are competent to help B cells develop into antibody secreting cells *in vitro*, in particular, those CD4 T cells expressing CXCR5 and PD1 and lacking CXCR3 (36). Are circulating CD4 T cells bearing these markers the primary source of memory CD4 T cells recruited into the lymph node after vaccination? It is known that CD4 T cells have considerable flexibility (64, 65), and it is possible that other subsets of memory CD4 T cells can develop helper activity during the response to vaccination. Also, because CD4 T cell-dependent antibody responses require B cell display of peptide:class II complexes, it is important to resolve if other viral proteins, particularly other membrane-associated proteins, such as M1 and NA, become co-internalized with HA by HA-specific B cells. If so, these proteins will also contribute peptide:class II complexes that can recruit additional Tfh. Similarly, because NA-specific antibodies elicited in response to vaccination are known to be a correlate of protection (66), it will be important to quantify the CD4 T cells that can promote production of these protective antibodies. Also, does antigen specificity influence the ability of CD4 T cells to convey the other functions of CD4 T cells in protective immunity to influenza? For example, does early vaccination and repeated antigen encounters, particularly with epitopes from highly conserved internal virion proteins, reinforce some functions of CD4 T cells that are associated with terminal effectors, such as cytolysis and gamma interferon production? These functions may not contribute to helper activity but may be important for other protective responses. Harty and coworkers (67) have shown through animal models of sequential encounter of CD8 T cells with antigen *in vivo* that the CD8 T cell transcriptome continually evolves with every “hit,” and we speculate that frequent infection or vaccination may promote a similar evolution in CD4 T cell function in humans.

**Figure 2:**
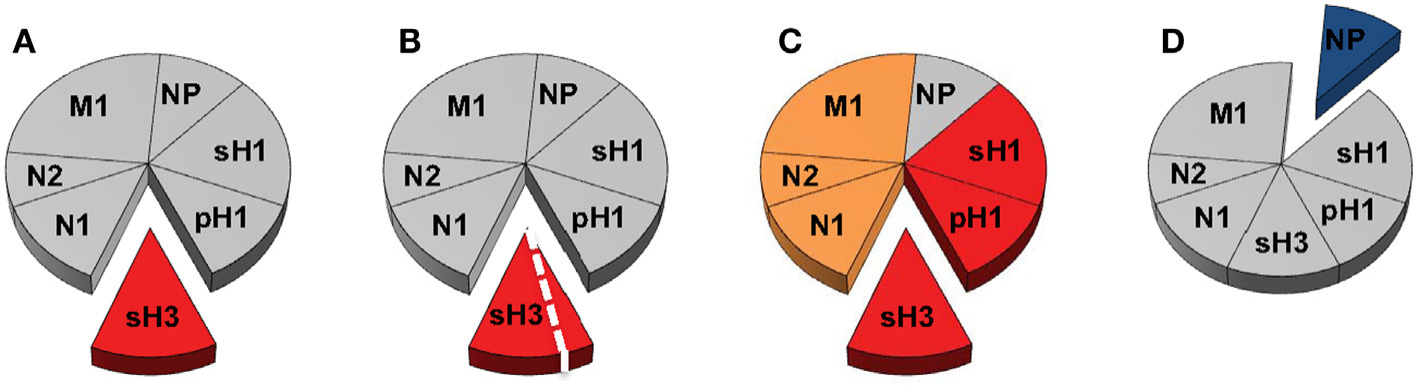
**Delineating the contribution of distinct CD4 T cell populations in protective antibody responses to influenza virus infection and vaccination**. The circulating human CD4 T cell repertoire against influenza is indicated in Figure 1 as a pie diagram with each slice representing relative reactivity to different viral proteins. Colors indicate speculated roles for CD4 T cells of differing antigen specificity with red representing known positive impact, orange unknown at present but possible, blue competitive or antagonistic impact, and gray indicating no impact. **(A,B)** represent CD4 T cell responses that may be relevant for neutralizing antibody responses after infection with an H3N2 virus, where those specific for HA may be the best correlate of help. **(B)** represents the possibility that CD4 help for production of neutralizing antibody may only be conferred by a subset of the HA-specific cells after infection, perhaps characterized by expression of CXCR5. In **(C)**, the possibility that after infection or vaccination, CD4 T cells specific for NA and M1, like those specific for HA may contribute to CD4 help for antibody responses because of their potential for physical interactions, leading to simultaneous uptake by HA-specific B cells. In **(D)**, NP (blue) is viewed as a potentially negative factor in the antibody response, although it may participate in an alternative CD4 function.

These questions are illustrated schematically in Figure 2, where we show the entire “pie” of memory CD4 T cells specific for influenza, where each slice represents a given viral protein specificity. We suggest that only some “slices” of this pie may be relevant to count toward productive antibody responses; HA being the most well documented thus far and NP being the most notably non-contributory. Because they are also membrane-associated, M1 and NA from infection or vaccines may be co-internalized with HA and CD4 T cells specific for these proteins may be able provide help, if these cells are contained within the CXCR5^+^CXCR3^−^ pools. Even within a given protein slice, there may be only subsets of cells that have the needed gene expression program and array of cell surface receptors to home to the lymph node and provide cognate help in the germinal center response. If this general model is correct, then there are only limited numbers of CD4 T cells that should be quantified for predicting future responses to vaccination and whose representation should be the goal of vaccination. Others may be neutral (in gray) and yet others may be a negative correlate of protection. At the same time, alternative subsets of cells may be the most relevant to mitigate the severity of an established infection, for example, those that express CXCR3, which may allow them to be selectively recruited to the site of infection. Here, their cytotoxic potential or secretion of cytokines may have an independent antiviral effect or promote viral clearance through the recruitment of other early effectors into the lung. Much progress by many groups has contributed thus far to our understanding of CD4 T cell function in influenza immunity. We suggest, moving forward, that more focused efforts to link these functions to CD4 T cell specificity, to consider both positive and negative effects of CD4 T cells and to utilize animal models with existing immunological memory for vaccine trials will together provide an increased accuracy in predicting the consequences of human encounter with pandemic viruses and vaccines. These new insights should provide the knowledge necessary to design the most effective prepandemic vaccination regimens that can optimally promote neutralizing antibody production against potentially pandemic strains.

## Ethics Statement

The University of Rochester Research Subjects Review Board approved this study protocol, and human experimentation guidelines of the US Department of Health and Human Services and the University of Rochester were followed. Study procedures were in accordance with the ethical standards of the Declaration of Helsinki, and all subjects provided written informed consent prior to participation in the study.

## Author Contributions

AS: wrote draft in consultation with all other authors and edited final versions. AD: literature review, assisted in writing draft, discussions, editing, figure design, and analyses. KR: provided experimental data, designed figures, and edited manuscript. ZK: literature review, editing, and discussions. JN: literature review, provided experimental data, figure preparation, editing, and discussion.

## Conflict of Interest Statement

The authors declare that the research was conducted in the absence of any commercial or financial relationships that could be construed as a potential conflict of interest.
